# Focal induction of ROS-release to trigger local vascular degeneration

**DOI:** 10.1371/journal.pone.0179342

**Published:** 2017-06-14

**Authors:** Jan-Philipp Minol, Isabella Reinsch, Maximilian Luik, Anne Leferink, Mareike Barth, Alexander Assmann, Artur Lichtenberg, Payam Akhyari

**Affiliations:** 1 Department of Cardiovascular Surgery, University Hospital, Dusseldorf, Germany; 2 Department of Tissue Regeneration, MIRA Institute, University of Twente, Enschede, The Netherlands; The University of Tokyo, JAPAN

## Abstract

Reactive oxygen species (ROS) play an important role in the process of cardiovascular degeneration. We evaluated the potential of a controlled, local induction of ROS-release by application of rose bengal (RB) and photo energy to induce atherosclerosis-like focal vascular degeneration in vivo. After injection of RB, rats fed with a pro-degenerative diet underwent focal irradiation of the abdominal aorta by a green laser (ROS group), while the controls received irradiation without RB. Aortic tissue was analyzed by histology and immunohistochemistry at 0, 2, 4, 8, 28 and 56 days (n = 5). The intimal surface topography was analyzed by scanning electron microscopy. In the ROS group, an initial thrombus formation had disappeared by day 8. Similarly, ROS-derived products displayed the highest concentrations at day 0. Relative matrix metalloproteinase (MMP) activity achieved a maximum after 8 days (ROS group vs. control group: 1.60 ± 0.11 vs. 0.98 ± 0.01; p < 0.001). After 28 days, no significant differences in any aspect were found between the ROS group and the controls. However, after 56 days, the aortic tissue of ROS animals exhibited relative media-pronounced thickening (ROS vs. control: 2.15 ± 0.19 vs. 0.87 ± 0.10; p < 0.001) with focal calcification and reduced expression of alpha smooth muscle actin (aSMA). The ROS-releasing application of RB and photo energy allowed for the induction of vascular degeneration in a rodent model. This protocol may be used for the focal induction of vascular disease without systemic side effects and can thereby elucidate the role of ROS in the multifactorial processes of vessel degeneration and atherogenesis.

## Introduction

A growing body of evidence supports the theory that reactive oxygen species (ROS) are a causative component in the pathophysiology of degenerative cardiovascular diseases, such as arterial hypertension [1], thrombosis [2] and atherosclerosis [3]. Particularly within the complex pathways of atherosclerosis, the discussed interactions of ROS are numerous. Oxidative stress impairs endothelial function, enhances smooth muscle cell (SMC) proliferation and inflammation and foam cell genesis by low-density lipoprotein (LDL) oxidation [4]. Moreover, vascular calcification can be promoted by oxidative stress-induced osteogenic transcription factors [5].

Most published models analyzing oxidative stress in the context of vascular degeneration and atherosclerosis in vivo have employed enzymatic or nutritive mechanisms [6,7]. These models also depend on systemically active factors with potential side effects on off-target organs.

Rose bengal (RB) (tetrachlorotetraiodofluorescein) is considered to be inert and to have low systemic toxicity [8] until it is exposed to green light with a wavelength of 543 nm [9]. When irradiated, RB produces singlet oxygen (^1^O_2_) [8,10] and, to some extent, superoxide-anions (O_2_^-^) [11] in a procedure, called photodynamic reaction (PDR). These species, in addition to hydrogen peroxide (H_2_O_2_) and hydroxyl radicals (HO^-^), are the prevailing components of the ROS family [12]. While this potential to form ROS species is well established, its application in the in vivo setting of animal models is mostly restricted to acute or short-term effects, e.g., focal thrombus induction [13] or damage of pre-existing atherosclerotic plaques [14]. Other groups focused on the potential to induce a non-mechanical endothelial injury to mimic adverse effects of cardiovascular interventions [15, 16]. These models fulfilled this purpose e.g. by demonstrating the generation of a neointima and its detailed analyses for follow-up periods of more than two months. However, to our judgment, the characteristics of the employed PDRs were aimed to initiate endothelial-focused, short-term and intense effects.

Contrary, we aimed to design the respective parameters of the PDR to avoid short-term effects as far as possible, which is in clear contrast to the design of previous reports. Instead, we rather aimed to ensure a long-lasting exposure of all vessel-parts to the PDR-induced ROS. Thereby, we analyzed the potential of the combined application of RB and laser energy to induce ROS for focal promotion of atherosclerosis-like vascular degeneration in an in vivo model

## Materials and methods

### Animals and dietary regimen

Male Wistar rats purchased from Janvier Labs^®^, France (n = 84; 200–250 g) were fed ad libitum with standard rat chow enriched with 300,000 IU/kg vitamin D, 1.5% dicalcium phosphate and 2% cholesterol beginning one week before the primary intervention. Five weeks after the beginning of the study (at t = 28 d), the diet was switched to standard chow to avoid exaggerated diet-caused calcification. The success of the pro-degenerative diet was controlled by analyses of serum for calcium, phosphate, cholesterol and triglyceride levels, obtained at euthanasia at t = 0, t = 8 d, t = 28 d and t = 56 d. These results were compared to the analyses of Wistar rats receiving standard chow (n = 5) and to reference levels.

All surgical procedures and animal experiments were performed in compliance with the Guide for the Care and Use of Laboratory Animals, as published by the US National Institutes of Health (NIH Publication 85–23, revised 1996) and were approved by the Committee on the Ethics of Animal Experiments of North Rhine-Westphalia („LANUV“), Germany; approval no.: 84–02.04.2011.A346. All surgery was performed under isoflurane anesthesia, and all efforts were made to minimize suffering.

### Induction of ROS

Rats were anesthetized by inhalation of 2.0–2.5% isoflurane. After Doppler examination, the abdominal aorta was exposed. A calculated amount of RB was injected into the exposed jugular vein (40 mg/kg body weight), and a laser beam (cw; 543 nm; 1 mW; 0.1 W/cm^2^) was targeted to the infrarenal aorta for 60 minutes. After the situs was closed in layers, another Doppler examination of the treated focus was performed, after which the animals recovered from anesthesia. For the control groups, the same procedure was performed without the application of RB. Postoperative pain management included subcutaneous administration of carprofen every 12 h for three days after surgery. Buprenorphine was administered once on the first day after surgery.

### Study design

Groups of n = 5 animals each were established to analyze the effects immediately after irradiation (t = 0), within a short-term period (t = 2 d, t = 4 d, and t = 8 d) and within a mid-term period (t = 28 d and t = 56 d). For each time point, five animals were treated with RB just before the laser was applied to induce ROS locally (ROS group), and five animals served as controls, receiving irradiation without the injection of RB (control group). For scanning electron microscopy, additional groups and their controls (n = 3 each) were established at t = 0; t = 8 d; t = 28 d and t = 56 d.

### Explantation and tissue analysis

Animals underwent laparotomy and euthanasia per exsanguination under general anesthesia. The section of the irradiated area of the aorta was explanted and opened at the dorsal side.

### Scanning electron microscopy

Specimens were fixed in a solution of 0.1 M sodium cacodylate, 2.5% glutaraldehyde (Polysciences, Warrington, PA, USA) and 0.2% formalin for 4 h. Afterwards, the specimens were dried using increasing concentrations of ethanol for 3.5 h and were further processed in a solution of 50% hexamethyldisilazane (HMDS) and 50% ethanol for five minutes before being immersed in pure HMDS for 10 minutes and overnight. The specimens were fixed on plates and coated with a 20-nm layer of gold in a sputter coater (108, Cressington, Watford, UK). Topographical examination was performed with a scanning electron microscope (XL 30 ESEM-FEG, Philips, Eindhoven, Netherlands).

### Histology and immunohistochemistry

Tissues were embedded in TissueTek^®^ mounting medium (Sakura Finetek, Alphen aan den Rijn, The Netherlands) at -20°C and were cut into 7-μm cryosections (cryostat CM 1950; Leica Biosystems, Wetzlar, Germany). For histological staining including Movat’s pentachrome and von Kossa, the protocols were applied as we described previously [17]. Imaging was conducted using a DM2000 microscope system with a DFC 425C digital camera (Leica Microsystems, Wetzlar, Germany) and Leica Application Suite V3.7 software.

For the determination of the relative thickness of the media by means of Movat’s pentachrome staining, an image-processing program (ImageJ, U. S. National Institutes of Health, Bethesda, MD, USA) was used. For quantitative analysis of the calcification based on von Kossa staining, the calcified area of each section was also determined in ImageJ. Similarly, the level of intensity of the 3-nitrotyrosine concentration within the aortic tissue was evaluated.

Immunohistochemical stainings were performed according to protocols that we described previously. We applied primary antibodies for caspase-3 (Abcam, Cambridge, UK), anti-von Willebrand factor (vWF) (DAKO, Hamburg, Germany) anti-alpha-smooth-muscle-actin (aSMA) (Sigma-Aldrich, Taufkirchen, Germany) and anti-3-nitrotyrosine (Abcam, Cambridge, UK). Secondary antibodies were conjugated to diaminobenzidine (DAB, Vector Labs, Peterborough, UK) and the fluorophores Alexa 546 and 488 (Invitrogen, Carlsbad, CA, USA) and Cy3 (Sigma-Aldrich, Taufkirchen, Germany). For immunohistochemical staining targeting caspase-3, hemalaun was applied as a counterstain. For fluorescence staining, the sections were covered with mounting medium containing 4',6-diamidino-2-phenylindole (DAPI; Vectashield, Vector Labs, Peterborough, UK). Image acquisition and processing were performed as described above.

### *In situ* zymography

For the determination of matrix metalloproteinase (MMP) activity, *in situ* zymography was conducted as described previously [17]. Cryosections of 7 μm were incubated for 24 h at 37°C in 40 μg/ml fluorescein-labeled gelatin (Invitrogen, Carlsbad, CA, USA) in 50 mM Tris-HCl supplemented with 5% Triton-X-100, 10 mM CaCl_2_ and 150 mM NaCl. Afterwards, Vectashield medium containing DAPI was used for mounting the sections. The MMP gelatinase activity was analyzed at 488 nm excitation. As a negative control, specimens were treated identically with additional supplementation of 20 nM ethylenediaminetetraacetic acid (EDTA). Quantification of the results was achieved by measuring the fluorescence activity at the focus of irradiation, which was normalized to the basal activity in non-irradiated areas.

### Statistics

Values are presented as scatter dot plots, representing the means +/- standard errors of the mean (SEM) (Prism5, GraphPad Software, La Jolla, CA, USA). Inter-group comparisons were performed by one-way ANOVA (InStat3, GraphPad Software, La Jolla, CA, USA).

For the analyses of calcium and phosphate serum levels, relative media thickness and relative MMP activity, the parametric Tukey’s post hoc test was applied. For analyses of tissue calcification, the nonparametric Dunn’s post hoc test was used. Probability values less than 0.05 were considered significant.

## Results

**Fig 1:**
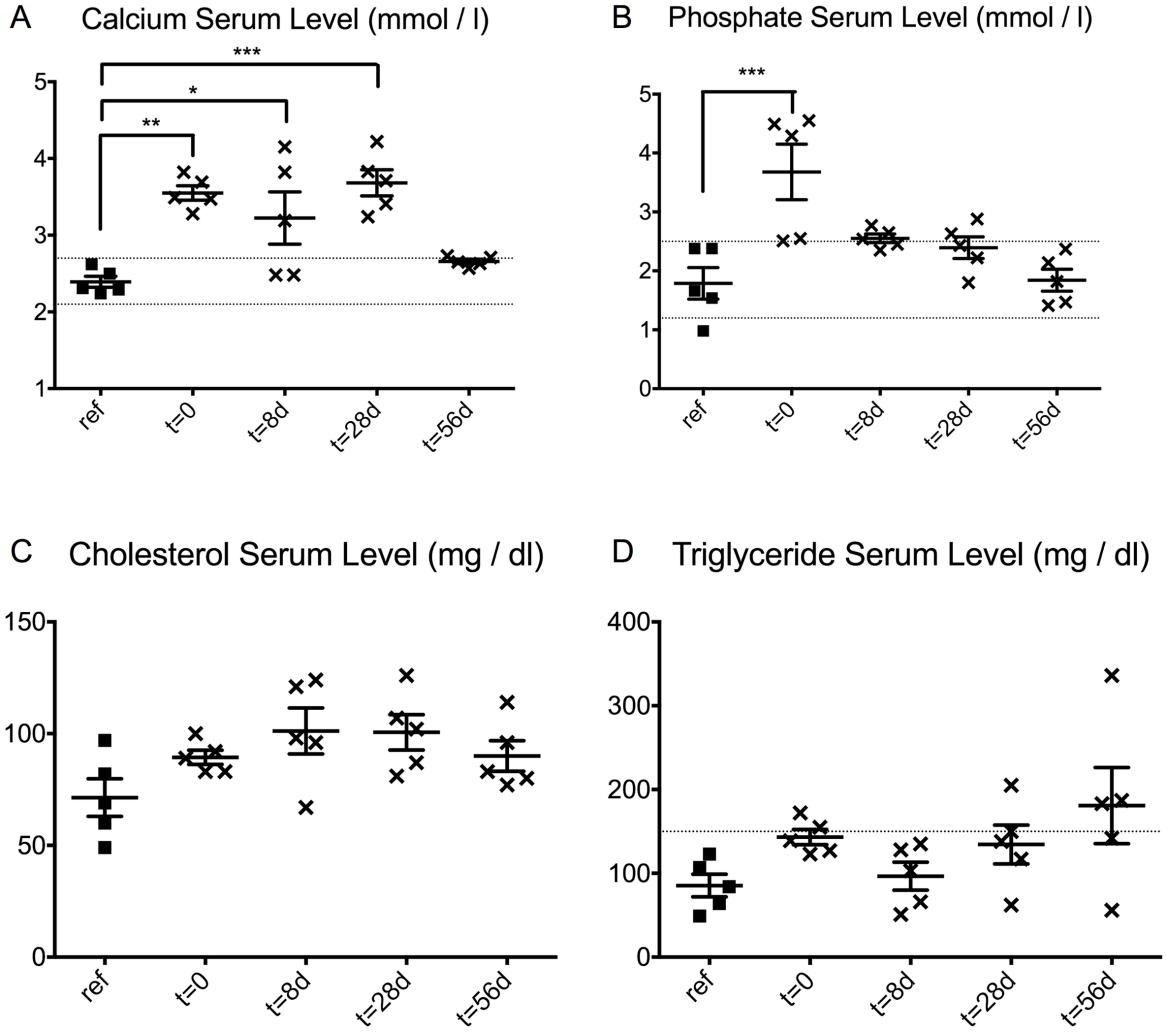
Effect of the pro-degenerative diet on serum levels. Serum analysis revealed the influence of the pro-degenerative diet with respect to levels of calcium (A), phosphate (B), cholesterol (C) and triglyceride (D) compared to physiological ranges (broken lines) and to a reference group (ref.). * p < 0.05; ** p < 0.01; *** p < 0.001.

The serum analysis of the reference group returned mean values for all examined parameters within the physiological ranges for calcium (serum calcium: 2.1–2.7 mmol/l; serum phosphate: 1.25–2.5 mmol/l; serum cholesterol: < 200 mg/dl; serum triglyceride: < 150 mg/dl) (Fig 1A–1D). Calcium serum levels were significantly higher in the diet-exposed groups at early time points, but this statistically significant difference was lost at the late time point of t = 56 d (Fig 1A). Phosphate serum levels were significantly higher than those in the reference group only at t = 0 (p < 0.001) (Fig 1B). Serum levels of cholesterol or triglyceride showed no significant differences from those in the reference group (Fig 1C and 1D).

Doppler examinations did not reveal any relevant impairment, e.g., accelerated or even discontinued blood flow at the treated focus at any time.

**Fig 2:**
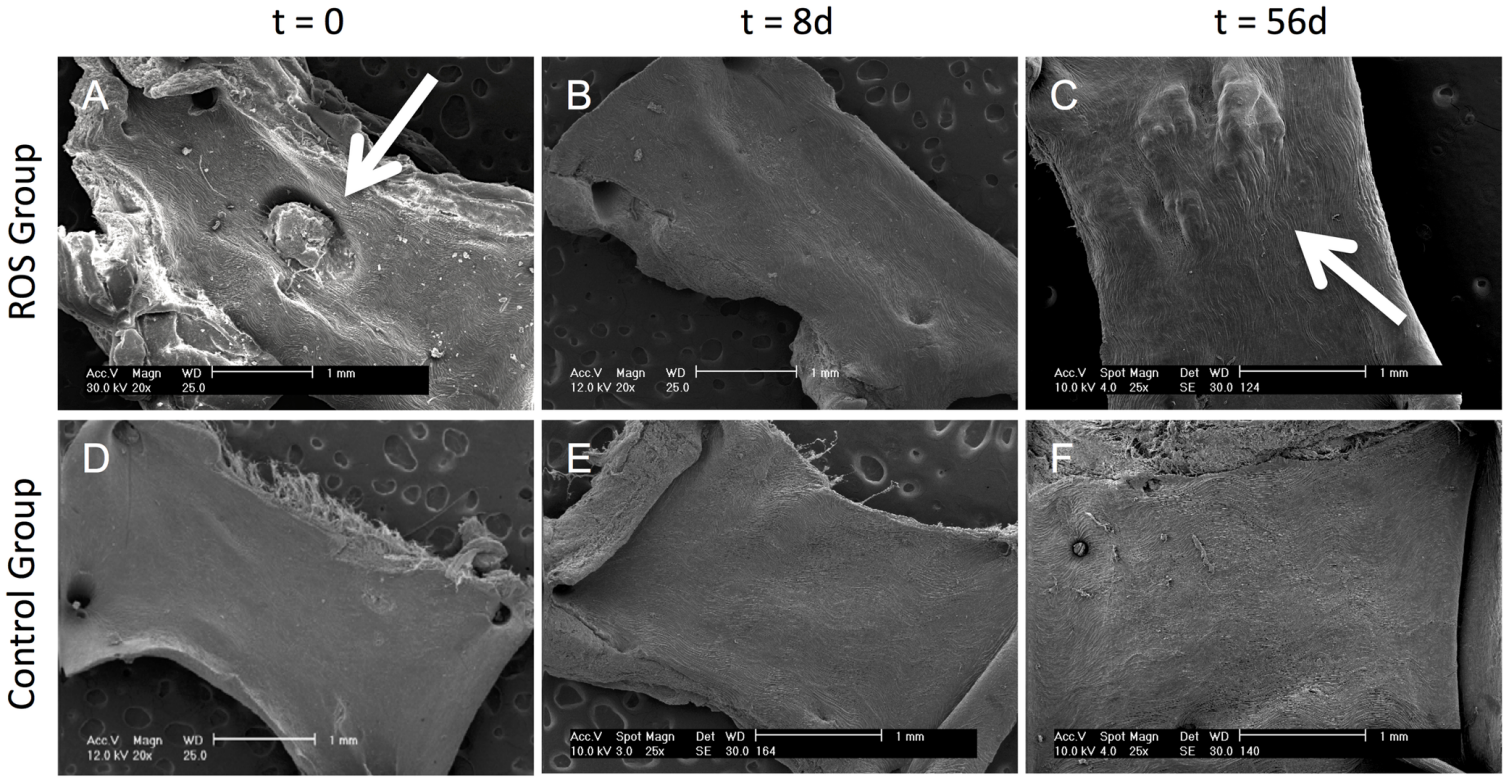
Analysis of the Endoluminal topography by SEM. The ROS specimens (Figs A-C) showed a thrombus at t = 0 (A; arrow) that was dissolved not later than t = 8 d (B). At t = 56 d, the ROS specimen exhibited the distinctive morphology of subendothelial wall thickening (C, arrow). In contrast, the controls did not display any changes at any time (Figs D-E). Scanning electron microscopy; scale bars = 1 mm.

SEM analysis of the aortic wall topography displayed in the ROS group a focal thrombus at t = 0 at the region of irradiation (Fig 2A; arrow). Later time points confirmed the disappearance of the thrombus by t = 8 d (Fig 2B), followed by the formation of an irregular sub-endothelial tissue prominence, elevating in the region of the irradiation into the aortic lumen at t = 56 d (Fig 2C; arrow). Rather, the control group did not display any changes at any time (Figs D-E).

**Fig 3:**
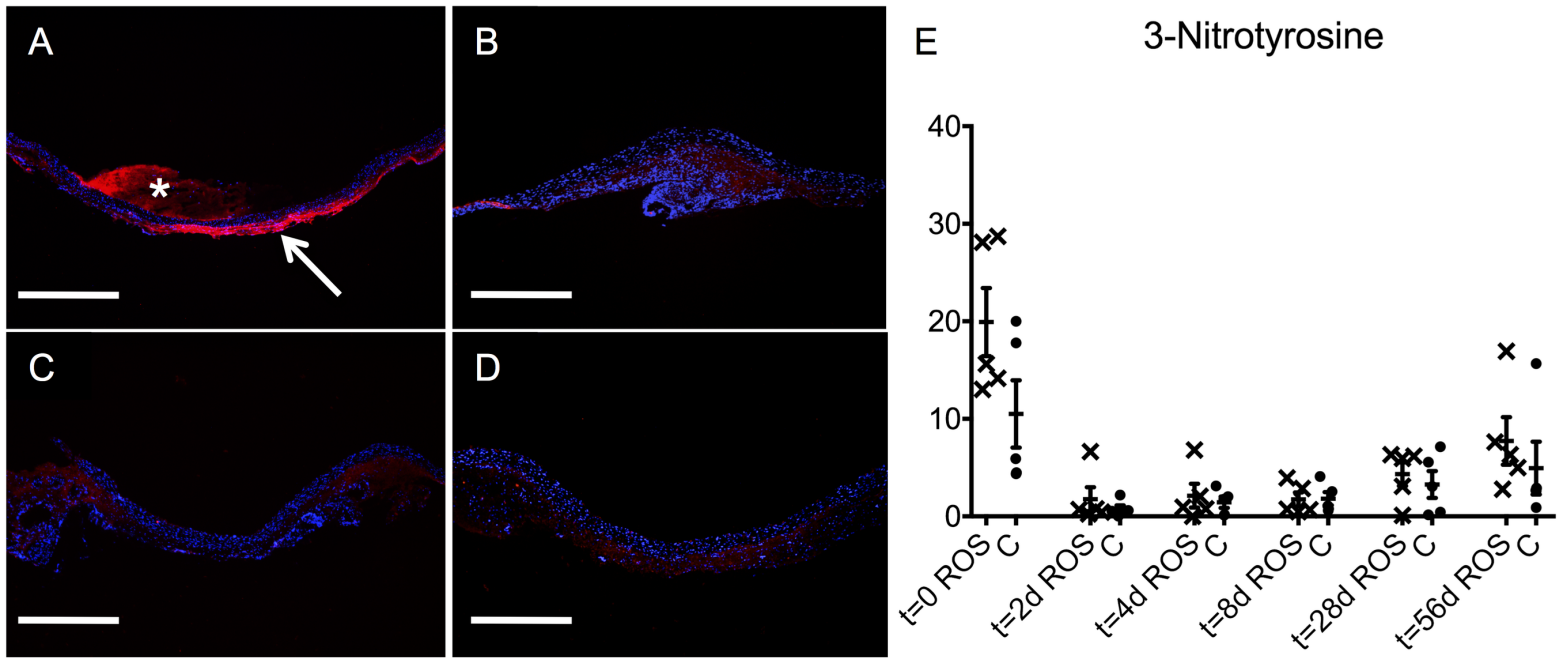
Indirect ROS-Detection by 3-nitrotyrosine intensity. Representative cross-section images displayed a distinctive intensity of 3-nitrotyrosine within the vascular tissue of the ROS groups at t = 0 (arrow in A) and to a reduced intensity at t = 56 d (B). Thereby, the most intense signaling was located in the outer part of the media and in the adventitia. Paralleled controls displayed only a slight intensity at t = 0 (C) and at t = 56 d (D), as well. 3-nitrotyrosin (red) with DAPI counterstain (blue); asterisk = thrombus; scale bars = 500 μm. (E) Semi-quantitative analysis of 3-nitrotyrosine activity in the vascular wall (0 = total darkness, 255 = maximum brightness; ROS = ROS group; C = control group).

The specimens were stained for 3-nitrotyrosine as an indirect marker of increased oxidative stress (Fig 3). This analysis displayed a focal intensity not only in the thrombus (Fig 3A; asterisk); but also within the vascular tissue of the ROS group at t = 0 (Fig 3A; arrow) and to a reduced intensity at t = 56 d (Fig 3B). The paralleled controls displayed only a slight intensity at t = 0 (Fig 3C) and at t = 56 d (Fig 3D), as well.

Semi-quantitative analysis of the 3-nitrotyrosine-fluorescence of the aortic tissue revealed the highest levels at t = 0 for both groups, with the maximum presented by the ROS group (Fig 3E). Subsequently, the intensity of 3-nitrotyrosine declined to a minimum at early stages and rose again at t = 28 d and t = 56 d in broad distributions in both groups.

**Fig 4:**
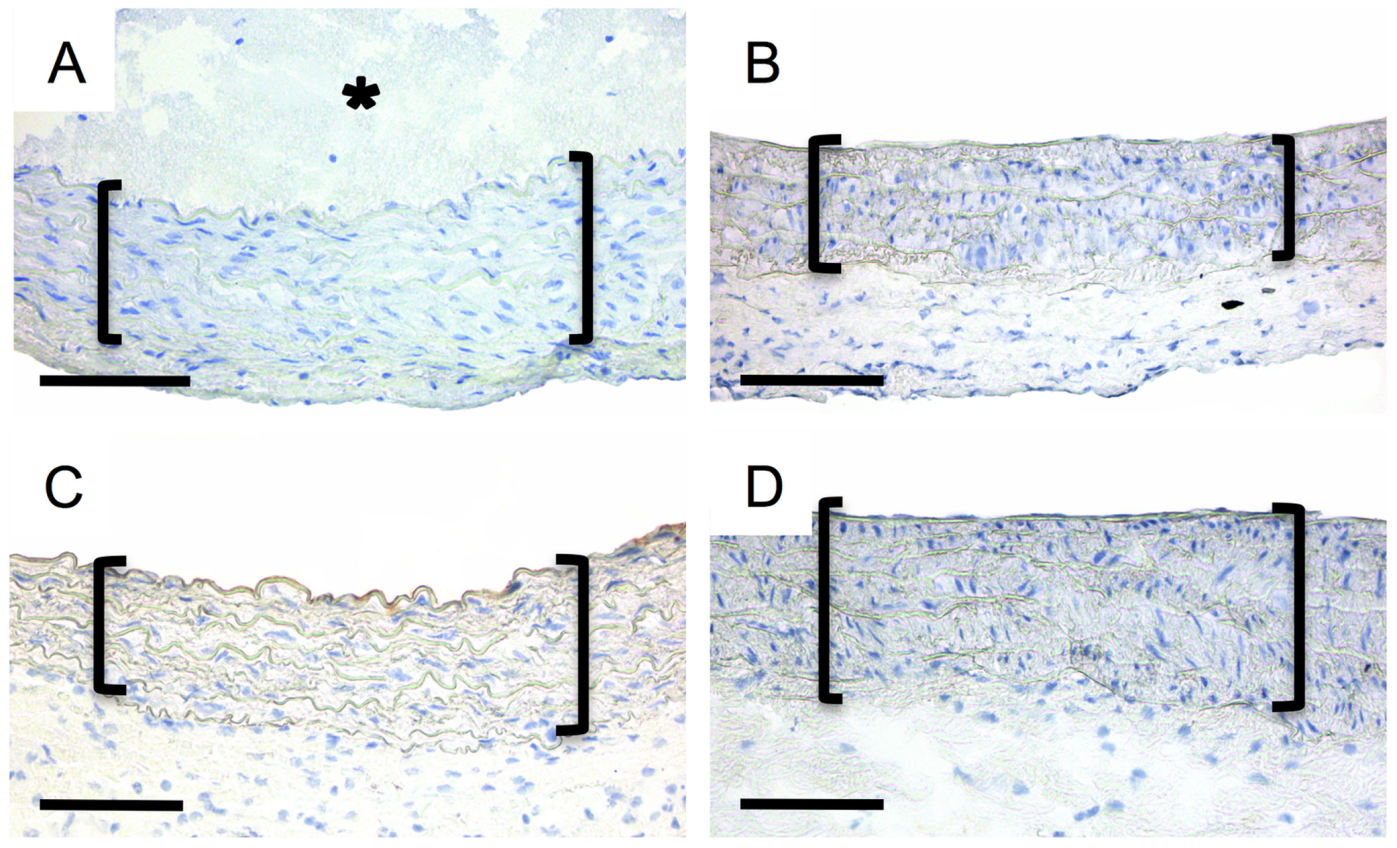
Analysis of the caspase-3 activity. Representative cross-section images of the ROS groups displayed no distinctive caspase-3 activity within the media (brackets) at t = 0 (A), t = 2 d (B); t = 4 d (C) and t = 8 d (D). Asterisk in (A) denotes the thrombus-formation. Immunohistochemistry: caspase-3 (DAB, brown) with hemalaun counterstain (blue); asterisk = thrombus; scale bars = 200 μm.

Apoptosis analysis via caspase-3 immunohistochemical staining did not reveal any enhanced intensity for ROS specimen (Fig 4A–4D).

**Fig 5:**
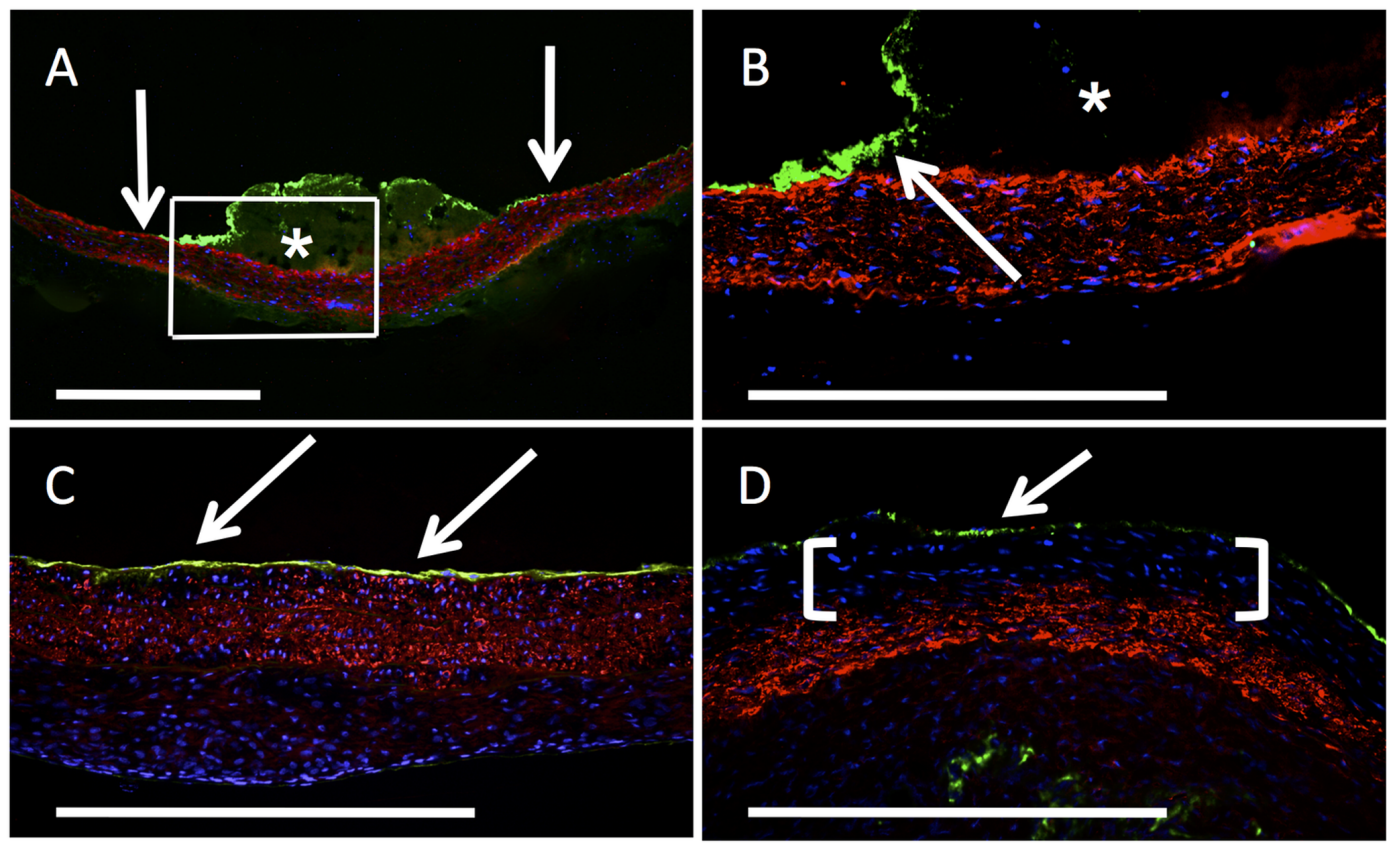
Analysis of the endothelial layer and aSMA expression. At t = 0 (frame in A is B), representative cross-section images of the ROS group displayed a continual endothelial layer (arrows) next to the thrombus (asterisk). The same focus displayed a continual layer after thrombus dissolution at t = 28 d (C) and t = 56 d (D). At t = 56 d (D); ROS specimen revealed a cell-yielding area with reduced aSMA expression (brackets), in contrast to the controls and earlier stages in the ROS group. Immunohistochemistry: vWF (green), aSMA (red) with DAPI-counterstain (blue); asterisk = thrombus; scale bars = 500μm.

Immunohistochemical staining for vWF revealed a fluorescent band in the region of the thrombus at the luminal surface in the ROS group at t = 0 (Fig 5A + 5B; asterisk), whereas the adjacent endothelial areas exhibited a regular luminal layer staining positive for vWF (arrows). After total thrombus resolution, vWF staining displayed a continuous vWF layer, indicating a closed endothelial layer as early as 28 d after irradiation (Fig 5C; arrows) and with essentially unchanged morphology at 56 days (Fig 5D, arrow). Detailed observation revealed a subendothelial area in the ROS group at 56 d, with cells of distinctively reduced aSMA expression in the region of the above-described wall thickening (Fig 5D; brackets). This finding was exclusively present in the ROS group and clearly contrasted with the findings from the earlier stages in the same group (Fig 5B + 5C).

**Fig 6:**
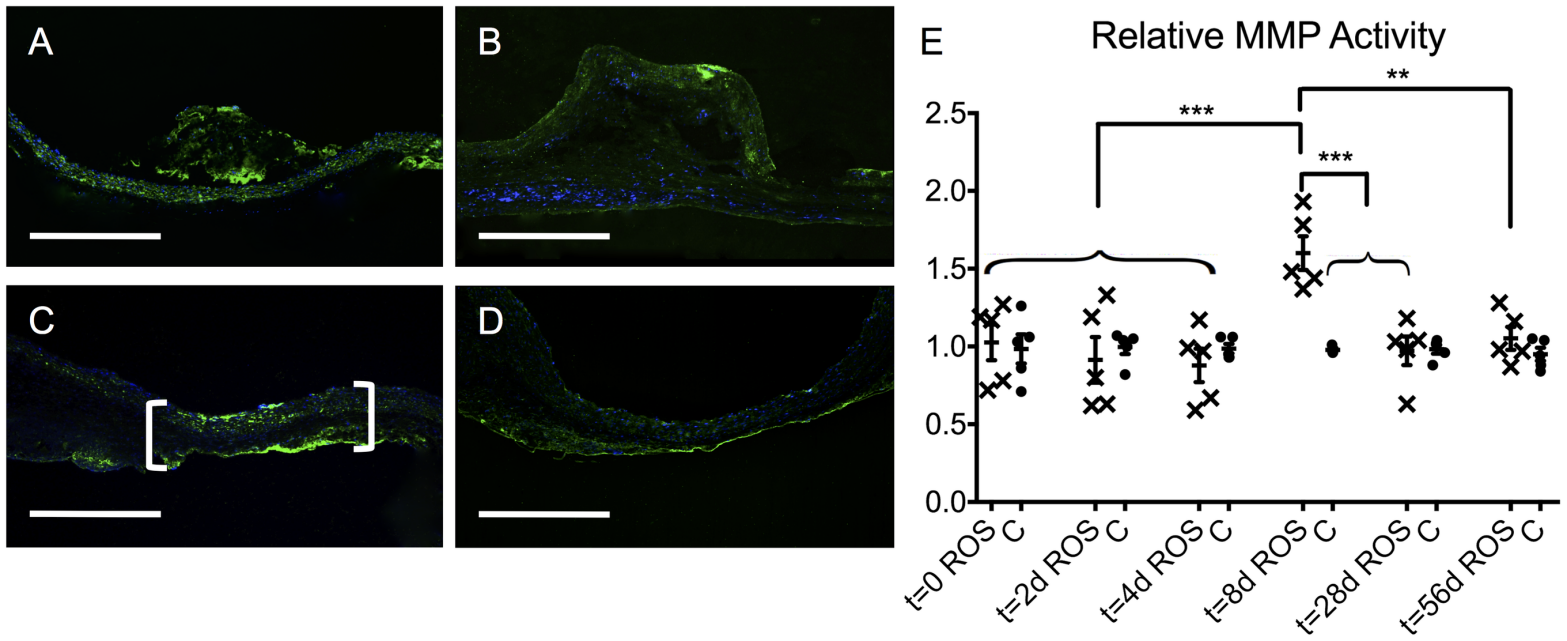
Analysis of the MMP activity by *in situ* zymography. Representative cross-section images displayed a homogenous MMP activity within the vascular tissue of the ROS groups at early stages such as t = 0 (A) as well as at late stages such as t = 56 d (B). In contrast, the ROS group at t = 8 d revealed a pronounced, focal MMP activity at the treated focus (C; brackets), whereas no comparable finding was to be noticed in its control group (D). *In situ* zymography: MMP activity (green) with DAPI-counterstain (blue); scale bars = 500 μm. (E) Semi-quanitative analysis of the relative MMP activity. ROS = ROS group; C = control group; ** p < 0.01; *** p < 0.001).

To characterize the dynamics of this process, an *in situ* zymography assay was applied to visualize the time-dependent MMP activity in ROS animals versus controls. In the ROS group (Fig 6A–6C), except for t = 8 d (Fig 6C) and in the control group (Fig 6D), MMP activity was only slightly distributed in the media and adventitia. In contrast, MMP activity was markedly increased in the area of irradiation of the ROS animals at t = 8 d (Fig 6C; brackets). Therefore, pronounced focal areas of intense MMP activity were detected in the adventitia and in the subendothelial region of the media compared to non-irradiated areas.

Semi-quantitative analysis of the relative MMP activity of the ROS group revealed a statistically significant increase at t = 8 d compared to that at all other time points (Fig 6E). Similarly, the MMP activity of the ROS group at t = 8 d was significantly higher than that in the control group at t = 8 d (p < 0.001).

**Fig 7:**
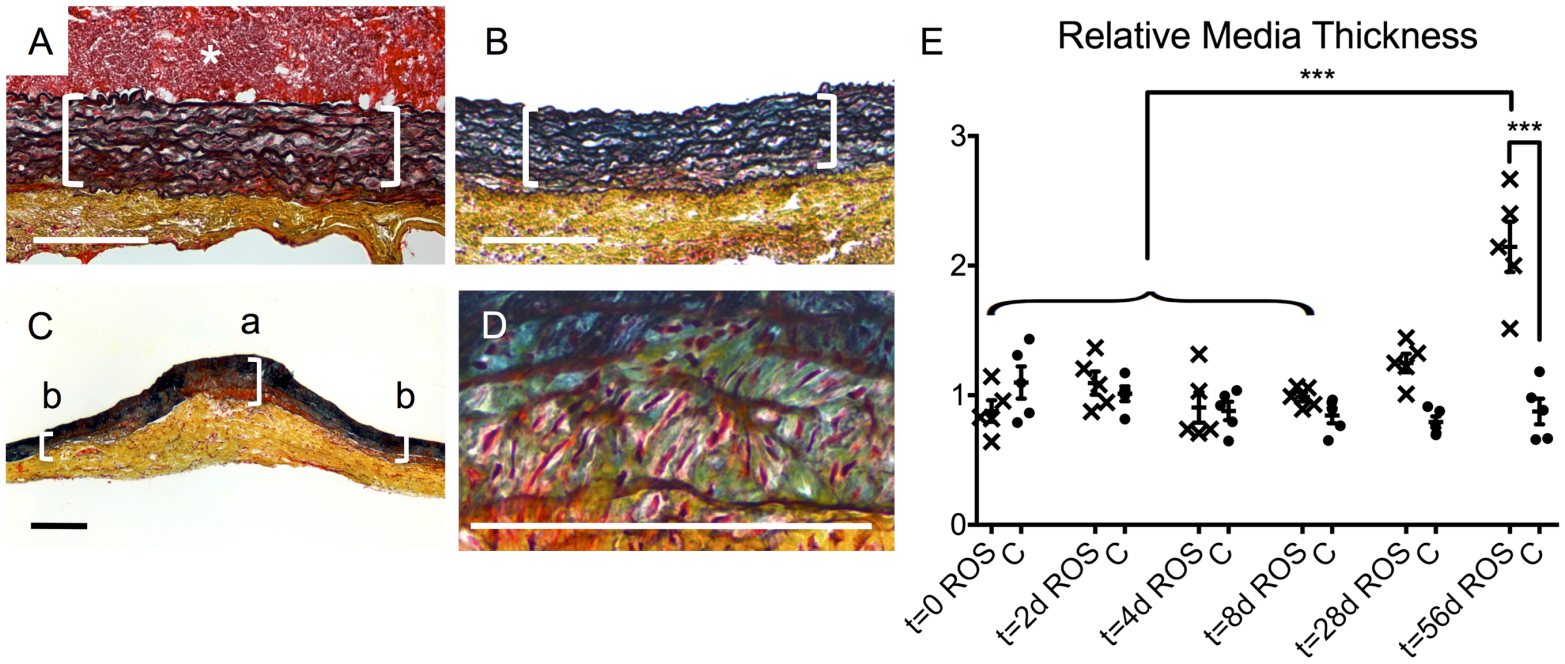
Histomorphological changes of the media. (A) A representative cross-section image of the ROS group displayed intact organization of closely spaced laminae of the media (brackets) at t = 0. The same appearance was found in the ROS specimens after thrombus-dissolution at t = 28 d (B) and in the controls at all times. (C) In contrast, ROS specimen showed at t = 56 d a distinctive, media-pronounced wall thickening at the treated focus (bracket “a”) compared to non-treated areas of the same specimen (brackets “b”). (D) A detailed analysis of this focus revealed a loosening of the laminae and enlarged amounts of green-bluish intercellular substance, consisting of high proportions of proteoglycans. Movat´s pentachrome staining: collagen (yellow), fibrin (red), proteoglycans (blue-green), elastin (black), nuclei (blue to black), cytoplasm (pink); asterisk = thrombus; scale bars = 200 μm. (E) Quantitative analysis of the media thickening at the irradiated focus. ROS = ROS group; C = control group; *** p < 0.001.

Movat’s pentachrome staining allowed further analysis of the tissue composition. At t = 0, the aortic wall area of the ROS specimens (Fig 7A) displayed at the treated focus under the thrombus (asterisk) an intact organization of the media with closely spaced laminae (brackets). Moreover, no changes to this region were found in either the ROS specimens after thrombus dissolution at t = 28 d (Fig 7B) or the control group specimens at all time points. Compared to the adjacent non-irradiated areas, the irradiated area of the aortic tissue of the ROS group showed a clearly broadened media at t = 56 d (Fig 7C). Detailed histomorphological analysis of this thickened area revealed a loosening of the laminae (Fig 7D). The intercellular substance displayed a blue–green colored staining, indicating high proportions of proteoglycans. For a quantitative analysis of the media thickening, the dimensions at the irradiated focus were compared to the adjacent non-irradiated areas (Fig 7C). This relative media thickening was statistically significant in the ROS group at t = 56 d compared to that at the earlier times up to 8 d and compared to that in the control group at t = 56 d (p < 0.001) (Fig 7E).

**Fig 8:**
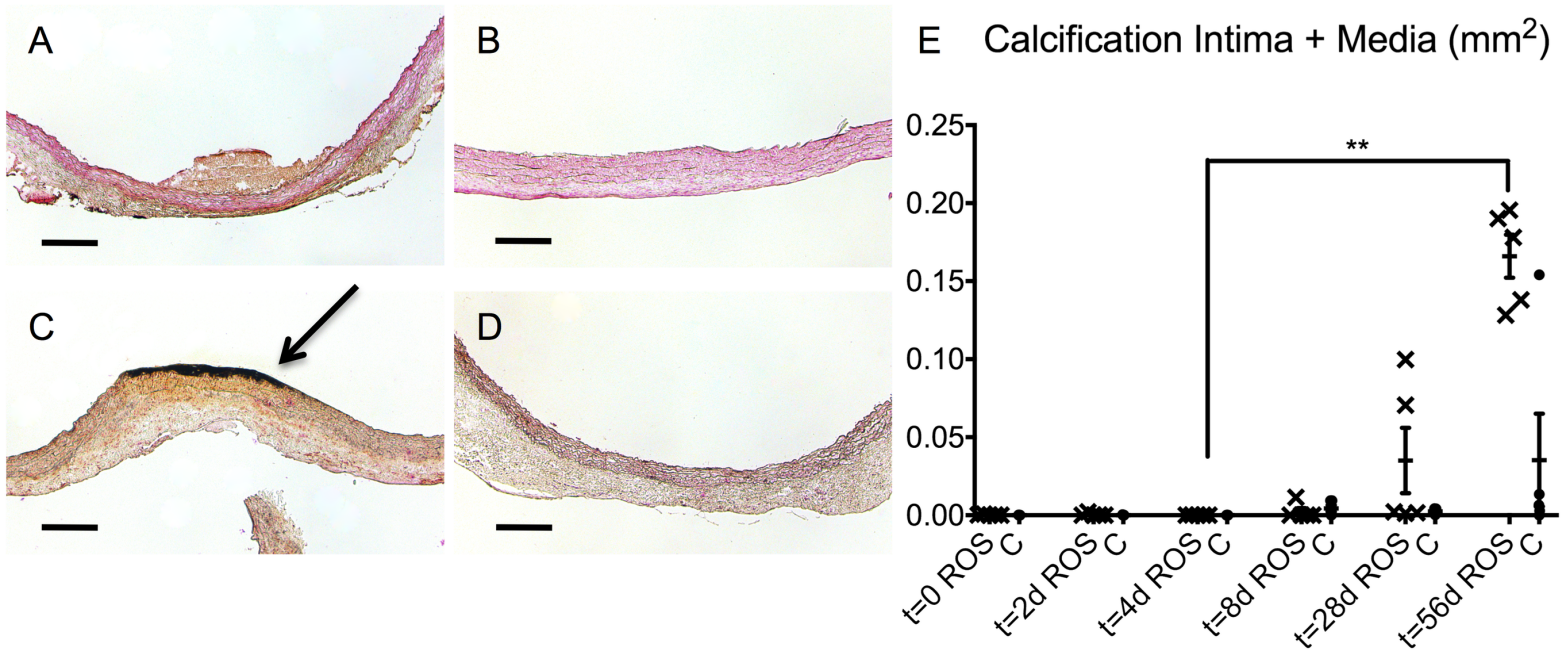
Analysis of the focal degenerative calcification. Representative cross-section images of the ROS specimen displayed no focal calcification at t = 0 (A) and up to t = 28 d (B). (C) At t = 56 d, a pronounced focal calcification (arrow) was noted at the lumen-facing side of the ROS-treated area. In contrast, conrol groups showed no focal calcification at t = 56 d (D) and earlier stages. Von Kossa staining: calcification (black) with nuclear fast red counterstain (red); scale bars = 200 μm. (E) Quantitative analysis regarding calcification of intima and media. ROS = ROS group; C = control group; ** p < 0.01.

Von Kossa staining was used to detect calcified areas in the aortic tissue. In the ROS group, no notable calcification was observed at the earlier stages of t = 0 or t = 28 d (Fig 8A + 8B), whereas at t = 56 d, a distinctive focal calcification in the area of irradiation was noted (Fig 8C, arrow). In contrast, no focal calcification was noticed in the controls up to t = 56 d (Fig 8D). Quantitative analysis of the calcified area of each slice displayed an increase in the ROS group that was only slight to moderate at t = 8 d and t = 28 d but was distinctive at t = 56 d (Fig 8E). This increase was statistically significant (p < 0.01) when compared to that in the ROS group at t = 4 d.

## Discussion

We demonstrated the feasibility of inducing ROS-mediated, focal vascular degeneration in vivo by a combination of RB and green laser energy. In this way, apoptosis and necrosis could be excluded as a key driving factors. ROS-exposed specimens displayed an initially enhanced intensity of 3-nitrotyrosine. An immediate, non-occluding thrombus formation was dissolved at t = 8 d, followed by a distinctive, media-pronounced wall thickening at t = 56 d after a refractory phase. Increased MMP activity at t = 8 d, followed by reduced aSMA expression in the media and wall thickening accompanied by tissue calcification, indicated the progress of tissue remodeling in the specimens in the ROS groups.

All animals treated with RB and laser light displayed immediate thrombus formation at t = 0, resolving within eight days after irradiation. This effect was supposed to be attributed primarily to impaired endothelial function or even injury [15, 16] and only secondarily to an activation of circulating platelets after irradiation of RB [18; 19]. However, neither scanning electron microscopy nor vWF-staining in the following points of time gave any indication of severe and sustained endothelial damage. No reactive generation of a neointima as described by previous studies could be observed [15, 16]. We noted an intact endothelium by SEM-analysis and vWF-staining that did not demonstrated any alterations compared to the controls 28 days after the PDR.

The staining of 3-nitrotyrosine displayed the most intense signaling located in the outer parts of the media and in the adventitia of the samples of the ROS-group at t = 0. As 3-nitrotyrosine is an indirect staining for ROS-derived products, its significance regarding a detailed analysis of the ROS-distribution within the vessel is limited. However, such finding by itself beside the existence of the thrombus in the lumen proves a relevant PDR that was present in all parts of the vascular wall.

The results of the hemodynamic analysis in our study contradict the findings of several previous studies describing an acute, total vessel occlusion by thrombi due to the application of RB and laser treatment. However, we have found no published description of a total occlusion of the abdominal aorta. Most reported occlusions occurred in vessels much smaller in diameter, such as carotid or femoral arteries [14–16]. To our judgment, the thrombus generated in our setting was large enough to have the potential to occlude these structures.

The distinctive wall thickening did not show a relevant impact on the hemodynamics with clinically apparent consequences. On the one hand, one could simply argue that the lesion was not large enough to cause a relevant hemodynamic impairment. On the other hand, this finding might be a result of the “Glagov” effect. Glagov et al. stated that the early stages of atherosclerotic lesions are paralleled by compensatory extension of the diameter of the artery [20]. Therefore, the luminal cross-sectional area is preserved for a long period and is impaired only at later stages. In future studies, one possible focus may be an in-depth study of cross-sectional aortic specimens to examine the luminal cross-section areas in detail.

The described media-accentuated wall thickening is paralleled by calcification with pronounced intensity in the intima and media of the irradiated region of the ROS group, particularly at t = 56 d. It is well known that high concentrations of ROS lead to increased cell death by activating elements of the apoptotic pathways, e.g., caspases [21]. This mechanism represents a main source of ectopic calcifications. However, our immunohistochemical staining for caspase-3 could not confirm a relevant level of apoptosis at any time. Necrosis as the prevailing type of cell death after physical or chemical injury should have been associated with an accompanying cellular inflammation. However, no hint for such one was to be noted in the other histological analyses. Therefore, we think that our approach in this exploratory study largely exclude initial cell death as prevailing source of the observed vascular degeneration.

Moreover, DAPI counterstaining did not suggest reduced cell contents in this calcified area. Rather, staining for aSMA in the area of focal vessel calcification indicated a loss of the smooth muscle-specific phenotype.

*In situ* zymography revealed a relative increase of MMP activity in ROS specimens at t = 8 d relative to that observed all other times in ROS groups and all control specimens. In vivo, MMPs are converted from proenzymes to the active forms [22], an effect that, along with the accompanying regulation of MMP activity, is related to tissue inhibitors of metalloproteinases (TIMPs) [23]. ROS are able to inhibit TIMPs and, thereby, to activate MMPs indirectly. This indirect pathway could be why elevated MMP activity was noted with a time delay of 8 d after exposure to ROS in our model. Studies by Rajagopalan et al. suggest SMCs as a source of MMP activity in our model. They demonstrated the capacity of peroxynitrite as a common side product of oxidative stress to activate cultured SMC MMP-9 [24].

Byon et al. described oxidative stress as an inducer of key osteochondrogenic factors that in turn promote a phenotypic switch of contractile SMCs to an osteochondrogenic phenotype [5]. Therefore, vascular SMCs contribute to the development of atherosclerotic lesions through increased migration, secretion of matrix components, osteochondrogenic differentiation, and the associated calcification [25]. Most of our results can be considered part of this causal nexus.

However, upon comparing our findings with the description of atherosclerotic lesions at different stages by Stocker and Keaney, with the exception of the wall thickening and focal calcification, several aspects were absent [26]. In our study, no signs of foam cells or a fibrous cap were noted. Moreover, no indications of significant SMC proliferation were observed.

Contrary to the models primarily focusing on an endothelial injury [15, 16], we modified the PDR by choosing a light source that was much less intense (1mW / mm^2^, 0.1 W / cm^2^) compared to that one e.g. employed by Kikuchi et al. (0.9 W / cm^2^) [16]. Our RB-concentration was two to four times higher than compared to the models of Kikuchi et al. and Hirata et al. [15, 16]. By choosing a large vessel like the aorta as the primary target, our protocol aimed at avoiding an occlusive effect by freshly formed thrombus material to assure a continual RB-supply to the irradiated area. The two latter aspects were aimed to allow a RB-diffusion into all parts of the vascular wall and an intact PDR throughout the duration of irradiation of 60 minutes. By all of these measures, we tried to avoid short-term effects as far as possible but rather to ensure an exposure of all vessel-parts to the PDR-induced ROS. These variations are most-possible causative for our different observations compared to Hirata et al. and Kikuchi et al. [15, 16].

At t = 28 d, we have not found any difference between the ROS-group and the controls, especially regarding the intact endothelium and the unaltered intima and media. This is in clear contrast to findings of Hirata et al. and Kikuchi et al., who consistently reported a pronounced neoimtima that achieved its final dimension three weeks after the thrombolysis [15, 16]. Moreover, they described an unaltered media, which was the focus of the major changes in our model.

## Conclusion

We established an animal model to analyze circumscribed aortic tissue remodeling and degeneration after focal induction of oxidative stress without systemic side effects. This model allows for the examination of ROS-mediated vascular degeneration via the comparison of ROS specimens and healthy vessel tissue from the same animal. This model supports the idea that oxidative stress is a trigger of SMC migration and transdifferentiation, along with biomineralization, and contributes to vascular calcification. However, further typical aspects of atherosclerotic lesions were absent, which indicates that ROS have an important role in the multifactorial processes of vessel degeneration and atherogenesis but may not be sufficient to induce all aspects of human atherosclerosis.

The comparison to previous models focusing on non-mechanical endothelial injuries could support our thesis of two different mechanisms: Most probably, initial short-term effects of the PDR cannot be excluded and are suspected to be responsible for the thrombogenesis. However, the observed intermediate phase at t = 28 d could be considered as caesura between initial direct effects and the results of the ROS-mediated pathways which are causative for our described media-pronounced alterations.

## Supporting information

S1 Table Minimal data set. (PDF)
