# Supplementary material for: Focal induction of ROS-release to trigger local vascular degeneration
Source: PLoS One. 2017 Jun 14;12(6):e0179342. doi: 10.1371/journal.pone.0179342 (PMC5470706; doi:10.1371/journal.pone.0179342)
Supplement: S1 Table — (PDF) [file pone.0179342.s001.pdf]

Minimal Data Set

Serum Analysis:

Calcium Serum Level (mmol / l)

ref.	t = 0	t = 8d	t = 28d	t = 56d
2.24	3.69	3.82	3.71	2.73
2.31	3.47	2.48	3.24	2.63
2.29	3.49	3.19	4.22	2.65
2.62	3.82	4.15	3.41	2.71
2.50	3.28	2.48	3.83	2.57

Phosphate Serum Level (mmol / l)

ref.	t = 0	t = 8d	t = 28d	t = 56d
0.98	2.55	2.77	2.63	2.37
1.66	2.51	2.65	2.22	2.14
2.38	4.49	2.54	2.88	1.47
1.54	4.55	2.45	1.80	1.82
2.38	4.29	2.35	2.43	1.41

Cholesterol Serum Level (mg / dl)

ref.	t = 0	t = 8d	t = 28d	t = 56d
49	92	121	102	96
60	83	98	87	114
82	89	96	107	77
69	83	124	81	80
97	100	67	126	83

Triglyceride Serum Level (mg / dl)

ref.	t = 0	t = 8d	t = 28d	t = 56d
49	172	51	205	56
107	123	66	138	183
123	127	128	62	336
64	139	103	117	187
84	155	135	150	142

3-Nitrotyrosine Activity (0 = total darkness, 255 = maximum brightness):

t = 0		t = 2d		t = 4d		t = 8d		t = 28d		t = 56d	
ROS	Cont.	ROS	Cont.	ROS	Cont.	ROS	Cont.	ROS	Cont.	ROS	Cont.
28.710	17.785	0.247	0.604	0.933	0.182	0.471	0.602	0.101	0.150	7.627	2.903
28.099	19.994	0.690	0.351	0.003	0.127	0.687	1.075	6.166	3.070	4.990	2.620
15.619	4.358	0.780	0.534	0.775	1.766	0.736	0.777	5.944	5.556	16.936	15.660
13.025	5.904	6.628	2.191	6.806	2.027	2.863	2.539	6.313	0.440	6.316	2.664
14.162	4.474	0.442	0.036	2.057	3.123	3.937	4.080	3.089	7.134	2.791	0.906

Relative MMP Activity:

t = 0		t = 2d		t = 4d		t = 8d		t = 28d		t = 56d	
ROS	Cont.	ROS	Cont.	ROS	Cont.	ROS	Cont.	ROS	Cont.	ROS	Cont.
1.270	1.060	0.800	1.000	0.590	1.060	1.370	0.960	0.630	1.010	0.970	0.940
0.780	1.030	0.630	1.040	0.990	0.930	1.930	0.970	1.030	0.880	0.870	1.040
0.720	0.860	1.330	1.070	1.170	0.950	1.480	1.010	1.040	1.020	1.280	0.880
1.190	1.260	0.620	1.050	0.970	1.060	1.440	0.970	0.980	0.960	0.980	1.050
1.170	0.710	1.190	0.820	0.670	0.930	1.780	0.980	1.180	1.040	1.160	0.840

Relative Media Thickness:

t = 0		t = 2d		t = 4d		t = 8d		t = 28d		t = 56d	
ROS	Cont.	ROS	Cont.	ROS	Cont.	ROS	Cont.	ROS	Cont.	ROS	Cont.
0.830	1.308	1.081	0.816	0.707	0.648	0.990	0.764	1.443	0.877	2.143	0.884
1.144	1.095	1.366	1.034	1.316	0.795	1.067	0.964	1.324	0.912	2.400	1.182
0.819	1.433	0.945	1.000	0.738	0.921	0.893	0.945	1.221	0.705	2.000	0.981
0.955	0.863	0.873	1.037	0.738	0.994	1.057	0.893	1.008	0.776	2.667	0.667
0.641	0.789	1.204	1.172	1.030	1.036	0.929	0.651	1.249	0.695	1.514	0.658

Calcification Intima + Media (mm^2):

t = 0		t = 2d		t = 4d		t = 8d		t = 28d		t = 56d	
ROS	Cont.	ROS	Cont.	ROS	Cont.	ROS	Cont.	ROS	Cont.	ROS	Cont.
0.000	0.000	0.000	0.000	0.000	0.000	0.000	0.009	0.100	0.004	0.178	0.013
0.000	0.000	0.002	0.000	0.000	0.000	0.000	0.009	0.002	0.002	0.190	0.154
0.001	0.000	0.000	0.000	0.000	0.000	0.000	0.000	0.002	0.002	0.138	0.006
0.000	0.000	0.000	0.000	0.000	0.000	0.000	0.003	0.001	0.004	0.128	0.003
0.000	0.000	0.000	0.000	0.000	0.000	0.012	0.000	0.071	0.000	0.196	0.000
